# Barriers and Enablers to COVID-19 Vaccination in San Francisco's Spanish-Speaking Population

**DOI:** 10.1007/s43477-023-00071-w

**Published:** 2023-01-11

**Authors:** Lucía Abascal Miguel, Canice Christian, Erin C. Accurso, Adriana Najmabadi, Priyanka Athavale, Jody A. Diala, Darpun Sachdev, Susan Philip, Michael J. Reid, Margaret A. Handley

**Affiliations:** 1 Institute for Global Health Sciences, University of California, 550 16th St, San Francisco, CA 94158 USA; 2 Department of Psychiatry and Behavioral Sciences, Weill Institute for Neurosciences, University of California, San Francisco, CA USA; 3 Department of Epidemiology and Biostatistics, University of California, San Francisco, CA USA; 4 School of Medicine, University of California, San Francisco, CA USA; 5 San Francisco Department of Public Health, San Francisco, CA USA; 6 Division of HIV, Infectious Diseases and Global Medicine, University of California, San Francisco, CA USA; 7 PRISE Center (Partnerships in Research in Implementation Science for Equity), University of California, San Francisco, CA USA

**Keywords:** COVID-19, Vaccination, Spanish speakers, Public health, Implementation science, Barriers, Access

## Abstract

Populations at high risk for COVID-19- including Spanish speakers—may face additional barriers to obtaining COVID-19 vaccinations; by understanding their challenges, we can create more equitable vaccine interventions. In this study, we used interviews to identify barriers and enablers to COVID-19 vaccine uptake among participants in the San Francisco Department of Public Health contact tracing program. Data analysis employed Capability, Opportunity, Motivation Behavior model (COM-B) and the Behavior Change Wheel framework as guides to target barriers with interventions and supporting policies. This paper presents data from interviews focused on COVID-19 vaccine uptake that was part of a project to improve COVID-19 preventive behaviors in San Francisco. We completed seventeen interviews between February and May 2021; six (35%) were completed in English and 11 (65%) in Spanish. Barriers to vaccine uptake included an unprepared health system, fear of side effects, limited knowledge, and conflicting information. Behavioral factors influencing vaccine uptake were mainly related to physical opportunity, automatic motivation, and psychological capability. Interventions that could address the most significant number of barriers included education, enablement, and environmental restructuring. Finally, communication and marketing policies that use diverse multi-lingual social media and environmental planning that includes accessible vaccine sites for people with disabilities, literacy barriers, and limited English proficiency could significantly increase vaccination. Public health departments should tailor interventions to high-risk populations by understanding the specific barriers they face. This exploratory study suggests how implementation science can provide frameworks to achieve this.

Despite widespread access to COVID-19 vaccines, the United States lags behind other high-income countries in vaccination coverage (Mathieu et al., 2021). The vaccination gap has impacted the country’s ability to fight the pandemic and new waves of infections due to arising new variants. Multiple barriers to vaccination have been described, including challenges with access, vaccine hesitancy, and lack of readiness. In the US, Black and Latinx people face more barriers than those who identify as White or Asian (Carson et al., 2021; Njoku et al., 2021). Recent surveys have shown that Latinx and low-income participants are more likely to wait to get the vaccine and less likely to trust public health officials regarding COVID-19 (Hamel et al., 2021).

As of December 2021, San Francisco, California, has had one of the lowest COVID-19 case and death counts of any metropolitan city in the United States (Kukura, 2021). Despite the overall success in mitigating the spread of COVID-19, the pandemic has highlighted striking disease disparities, disproportionally affecting racial and ethnic minorities. This pattern is like what was observed in the rest of the country, where Black and Hispanic or Latinx persons and Native Americans had higher rates of infection and hospitalization than non-Hispanic White persons (Acosta et al., 2021). Factors including increased exposure, limited access to information, and limited and differential access to healthcare services, including COVID-19 diagnostics and care, are some of the challenges these communities have faced throughout the COVID-19 pandemic in the United States, including San Francisco (Thakur et al., 2020; Unruh et al., 2022).

Hispanic or Latinx people comprise 15% of San Francisco’s total population but account for 35% of all its COVID-19 cases (City and County of San Francisco, 2021). The disparity in case rate was more significant in the first year of the pandemic; from April to June 2020, when 70% of COVID-19 cases and their close contacts participating in the city’s contact tracing program were Latinx and of these 85% reported Spanish as their primary language (Sachdev et al., 2021). This project was initiated in response to the San Francisco Department of Public Health’s (SFDPH) need to understand adherence to Public Health recommendations within their contact tracing program among Spanish speakers, who were overrepresented in their cases and contacts (Sachdev et al., 2021). The City of San Francisco partnered with UCSF implementation scientists to understand the specific barriers this group faced to prevent infection and later, as vaccines became available, increase vaccine uptake within this group.

There is strong evidence that a targeted behavioral approach is needed to design strategies to successfully change behavior and increase adherence to public health measures (Eaton & Kalichman, 2020; Evans & Bufka, 2020). This need has been even more apparent with the COVID-19 pandemic, which has required individuals and communities to act and navigate various behaviors, including vaccination. Public health systems must first understand the barriers and enablers that specific communities face to design better vaccine interventions. Vaccination strategies that consider human behavior must focus on structural, systems, and socially based drivers of risk and inequity.

We applied a theory-informed assessment to aid the selection of evidence-based intervention strategies to improve uptake of approaches to increase vaccination among the primarily Latinx population in San Francisco, focusing on individual and contextual factors. For this, we used the Capability, Opportunity, and Motivation Behavior (COM-B) Model and related Behavior Change Wheel (BCW) framework to identify specific barriers and enablers in this population and guide the selection of interventions and policies to address them (West et al., 2020). This paper illustrates one approach for how public health departments can use implementation science frameworks, such as the COM-B model, within their programs to better inform their design by considering the needs of high-risk populations. The objective of the study was to aid the SFPHD in identifying barriers and enablers contact traced participants were facing in ‘real-time’ as the pandemic unfolded (shelter in place, masking, vaccination). To build on understanding these barriers, the project also focused on identifying intervention functions and policies to address them. Additionally, this paper seeks to illustrate how implementation science models and frameworks, specifically the COM-B model and BCW framework, can be applied in real-time in public health programs to identify problems and find solutions.

## Methods

### Setting and Study Design

We conducted a prospective observational qualitative study within the contact tracing (CT) Program in San Francisco, administered by the San Francisco City and County Department of Public Health (SFDPH). The study was iterative and designed to adapt to evolving priorities set by the SFDPH. Initially, the 1-year study focused on identifying barriers to behaviors around self-isolation, quarantine, and testing. As vaccines became available, the study pivoted to better understand barriers and enablers to COVID-19 vaccination. This paper includes the subsample of interviews done after COVID-19 vaccines became available. The study was a collaboration between SFDPH and the University of California, San Francisco (UCSF) researchers.

We recruited participants from the San Francisco Department of Public Health's Contact Tracing Program (SFDPH CT) between February and May 2021. We used purposive real-time sampling to find people that recently participated in contact tracing activities using bi-weekly random sampling among COVID-19 contacts, oversampling Spanish speakers to achieve a 2:1 ratio to reflect the demographics of San Francisco’s COVID-19 case burden at the time. Eligibility included people over 18 who had been exposed in the last week to COVID-19 and were still in quarantine and spoke Spanish or English. We included one participant per household or cluster to increase diversity of the sample and reduce the risk of duplication of findings.

After the SFDPH CT team completed an initial contact tracing call, which reached over 80% of reported COVID-19 exposures, those sampled received a follow-up call from a language-concordant research team member (Sachdev et al., 2021). The interviewer described the study and asked for consent to participate in a one-hour in-depth interview. Interviews were conducted by phone, recorded, transcribed, and translated (for interviews conducted in Spanish). The study team also prepared interview memos following each call. The SFDPH research review committee and the UCSF Committee on Human Research (IRB# 20-31,634) approved all study procedures.

### Theoretical Approach

We applied the Capability, Opportunity, and Motivation Behavior (COM-B) Model and related Behavior Change Wheel (BCW) as the guiding framework to help us better understand the population and individual behaviors relevant to COVID-19 prevention within each person’s specific context. The COM-B model and BCW were developed as a synthesis of nineteen behavior change frameworks identified through a systematic review (Michie et al., 2005). The BCW framework uses the COM-B model at its center to identify barriers and enablers to targeted behavior, such as getting vaccinated, in context. COM-B specifies that to change behavior, individuals need to be able to change or have the environment around them support change. Specifically, the framework helps identify whether Capability, Opportunity, and Motivation-related factors drive a specific behavior. For any given behavior, a person needs the ‘capability’ to perform it, including skills, knowledge, and physical strength; the ‘opportunity’ in terms of the physical and social environment, affordability, accessibility, and social support; and lastly, they must be ‘motivated’ to complete such behavior. Once we identify barriers and enablers, the next steps of the BCW provide guidance to identify intervention functions and their supporting policies to address the behavioral barriers and leverage enablers identified through COM-B, thus creating a ‘road map’ for intervention designs. The BCW framework provides a basis for translating stakeholder input into interventions that change the desired behavior (Michie et al., 2013, 2014). For this project, we used COM-B to (1) develop the interview guide and survey; (2) code transcripts and conduct thematic analysis; and (3) prioritize modifiable barriers and enablers for intervention targeting. We then used the BCW to identify a list of intervention functions and supporting policies mapped to the identified barriers and enablers.

### Interviews

Using COM-B conceptual model, we developed an in-depth semi-structured interview guide that incorporated findings from an initial assessment of barriers based on previous results (Thakur et al., 2020). The iterative guide initially asked about COVID-19 prevention barriers and enablers, focusing on behaviors recommended by the CT program and the socio-economic context that contacts were facing (shelter in place, masking, return to work) as they attempted to adhere to recommendations and shifted to ask about vaccines as they became available. This paper reports on those who were included in the vaccine-specific interviews, including behaviors and intervention components that could encourage COVID-19 vaccine uptake. The study sample reflects the composition of the contact tracing program participants at the time of the study. The interviews included questions about motivational barriers, such as beliefs and fear of the vaccine; capability barriers, such as skills related to scheduling and navigating the vaccination process; and opportunity barriers, such as asking about social norms and the influence of peers on their decision to get or not vaccinated against COVID-19.

Additionally, we also asked them to provide personal recommendations on what would improve vaccine access in their communities, their perceptions of the SFDPH COVID-19-related programs, and the role of community-based organizations (CBOs). The structured part of the survey was completed in REDCap by the interviewer; interviews were recorded with prior consent, transcribed, and translated to English if conducted in Spanish. Participants received a $25 e-gift card for their participation.

### Data Analysis

We analyzed transcripts concurrently with data collection to have a real-time feedback mechanism that included sharing results with the SFDPH CT program in reports and presentations. All transcripts were analyzed to identify perceived and experienced vaccine-related barriers and enablers. We based data analysis on applied qualitative inquiry (Sandelowski, 2004). A priori codes were determined using the COM-B model, and all transcripts and memos were coded by two independent reviewers using Dedoose version 7.0.23. The coding team had high inter-rater reliability (> 80%), as calculated by Dedoose after the coding of 5 initial transcripts through the program's "Training Center," in the analysis planning team meetings. A study team comprised of the primary investigator and three co-investigators met weekly and reviewed findings.

Once we identified what needed to change to increase vaccine uptake through our COM-B behavioral analysis, we used the BCW framework as a guide to identify intervention functions and supporting policies that would be effective against the identified barriers. We used an alluvial chart to graphically depict how barriers link to intervention functions and their supporting policy categories. Our depiction is similar to the wheel the BCW framework uses to show what intervention function and policy categories can be used to address specific COM-B categories (Michie et al., 2013).

## Results

### Participants

We completed 17 interviews specific to COVID-19 vaccine uptake barriers and enablers between February and May 2021. Eleven (65%) of the interviews (Table 1) were done in Spanish and 6 (35%) in English. Three participants who completed the interview in English were also Spanish speaking but preferred English. Overall, most participants identified as female; male and female participation in the English interviews was equally split, while 72% of the Spanish interviews were among females. The mean age for participants was higher for Spanish interviews than for English (41 vs. 36 years, *p* < 0.001). Fifteen (88%) of our participants identified as Hispanic or Latino, and 2 (12%) identified as White. All the Spanish-speaking participants lived in zip codes in which the estimated median household income is lower than San Francisco’s median household income, which was $119,136 in 2020. Of the participants that qualified for the vaccine (*n* = 13), ten had received at least one dose of the vaccine. We did not follow-up to ask about vaccination status at a later point in time.

**Table 1:** Sociodemographic characteristics of participants

Participant characteristic	Full sample		Spanish		English	
	*n*	%	*n*	%	*n*	%
Completed interviews	17	100	11	65	6^a^	35
Gender						
Male	6	35	3	18	3	18
Female	11	65	8	47	3	18
Age (mean, SD)	39 (13.32)		41 (12.02)		36 (16.13)	
Race and Ethnicity						
White	2	12	0	0	2	12
Hispanic or Latino	15	88	11	65	4	24
High risk for severe COVID-19^b^	6	35	4	24	2	12
Household size (mean, SD)	4.5 (3)		4.6 (3.82)		4.3 (2.41)	
Zip code by median income						
First quartile	6	35	6	35	0	0
Second quartile	0	0	5	29	3	18
Third quartile	2	12	0	0	2	12
Fourth quartile	1	6	0	0	1	6

Zip codes in San Francisco were ordered by median income based on the most recent US census data and divided into quartiles. Median household income quartiles are identified from first to fourth, indicating the poorest to wealthiest populations

^a^Three were bilingual but preferred to be interviewed in English

^b^Patients 65 or over or with a preexisting condition that increases the risk of COVID-19 hospitalization or death

### COM-B Barriers and Enabler Themes

We categorized key themes from the analysis using the COM-B model (Table 2). We identified (1) perceived system-level barriers, including poor systems preparedness and a lack of coordination between system players; and (2) individual-level barriers that reflected a wide range of beliefs and experiences, from confusion and lack of clarity about vaccine eligibility to fears of side effects or government control. Systems barriers fell into the COM-B category of *opportunity* barriers and were reported more frequently in the early phases of vaccine roll-out. Participants mentioned the system was unprepared to provide vaccines, for example, by having strict eligibility criteria that confused who qualified and when and perceived a lack of communication and coordination between vaccine providers. On the individual level, the main *capability* barriers were knowledge about the safety and side effects related to the vaccine and limited skills to gain such information.

**Table 2:** Main barriers and enablers for COVID-19 vaccination by COM-B category and theme

COM-B category and theme (*N*)	Example quote
Capability barriers	
Poor or limited understanding of the safety and effectiveness of the vaccine makes people hesitant. (*N* = 9)	"I don't even really trust the vaccine just because how could you have a vaccine for something that you don't know… that you don't know where it came from? But you don't have a vaccine for HIV, AIDS, you don't have a vaccine for cancer, lupus, none of vaccines for none of these other things but you have a vaccine for COVID-19. And then it's like, what's the purpose of the vaccine if you can still catch COVID? So basically, I'm injecting some foreign object inside me because I don't know what it is, you are injecting something inside of me because you feel like it's the vaccine for COVID. But if you don't know where COVID is from, how can you make a vaccine for it? You can't."
Conflicting information from different sources creates confusion and hinders the ability to decide on the vaccine. (*N* = 6)	"Things that I hear in the news about the vaccine are confusing. They say one thing and then another. Honestly, I don't know what to think."
Capability enablers	
Community-based organizations play a vital role in encouraging and facilitating vaccination. (*N* = 9)	"I went to 18th and Shotwell; one of my friends gave me this info. He works in a CBO. I was looking for an appointment close to me, but there weren't any. I was worried because they weren't any appointments for this year. It was saturated. So, I went to where the CBO told me, so I just walked there, and everybody was super nice. I didn't even have to wait. It didn't even hurt. I waited there for 15 min, and everything went great. People were amazing. I was expecting a more complicated experience with longer lines.”
A more nuanced understanding of the vaccine increases confidence in vaccination decision-making. (*N* = 17)	“I think that the way it goes, that it's about not getting COVID. I might still get it, but it won't kill me. So that's what I understand from the vaccine. I don't think it makes me like immune to it, that I'll never get COVID, I think there is a very good a chance I could still get it, but especially if I go out and not everyone is vaccinated, but because of that, it won't kill me.”
Opportunity barriers	
The lack of clear communication between health systems, providers, and patients created confusion around vaccine eligibility, appointments, and roll-out. (*N* = 6)	"The hardest thing is trying to find out about the vaccines. You know, I mean, in SF it's so chaotic. No one knows what they're doing. You can't get an answer. They kind of blew me off. Said that I needed to wait and pay attention to the news.”
A lack of coordination within the system complicates navigation and access. (*N* = 17)	“Most of the time people are going to be working 8–12 h shifts and hard to go through loopholes and going to inconvenient places and deal with people if they're rude.”
Opportunity enablers	
Identifying as part of a group or religion that is pro-vaccine makes people confident and vocal about others being vaccinated. (*N* = 8)	“I was able to do it through the organization "Excelsior Strong." It makes a big difference to have organizations help. I belong to an Aztec dance group, and a member was able to schedule all the Elders of the group to get vaccinated. Around 10 of us went.”
Vaccine outreach from CBOs, clinics, or health departments increases the completion of vaccination. (*N* = 6)	“When my parents were eligible for the vaccine they got notified via text, which made things easier. I would like that as well.”
Motivation barriers	
People are afraid of long-term side effects and permanent changes to the body. (*N* = 14)	“I plan on getting it in 2 years. I gave it 2 years to see how people's bodies react, because you know people are saying on the second shot, they are getting a little cold maybe, they have a little cough, a headache, or a little fever maybe, but that's only because they only get that after that second shot. But what's going to happen after 2 years? How is the shot going to affect your body then? I would rather just give it a time period, so I see how it’s going affect people's bodies. I know more than 20 people who have it, so I'm going to see this how going to affect their bodies.”
Legitimacy Concerns: Vaccine is new and not well tested yet, mistrust in government. (*N* = 9)	“I feel like the roll-out of the vaccine was rushed, I just don’t how effective it would have been if we could have waited a little longer or put more time into making the vaccine. For example, the J&J was recalled; they had to recall a certain lot for Moderna, etc. I know that they had to rush it because of the severity of the pandemic, but I always wonder if they had more time or did things a little more different. I’m hesitant and scared of the agenda behind the vaccine and why it's being pushed.”
Motivation enablers	
A trusted person and community setting are motivating for vaccination (*N* = 12)	“The Priest in the church I go to is very involved and has talked a lot about the virus, they've used the church for testing.”
Fear of getting COVID inspires vaccination (*N* = 7)	“I'm concerned about getting covid. Once I get vaccinated, the side effects are worth it. I had family members in Los Angeles who passed away due to COVID or got really ill, which made me really want to get it.”
Getting the vaccine is a positive commitment to friends, family, community (*N* = 13)	“It's important to protect the most vulnerable community.”

*N* number of participants that identified each barrier

In many cases, participants referred to social media/networks to fill in gaps rather than health systems providers. Many participants did not know how the vaccines work, how they were produced, and how the government regulated the approval process. Some participants lacked the skills to seek information or schedule an appointment online due to low general and tech literacy. A consistent theme was the role of social media as a source of information and misinformation. Health concerns that reduced *motivation* for vaccination included fear of immediate and long-term side effects and worry that the vaccine would not protect against new variants. Some participants cited media stories about side effects such as blood clots and myocarditis. Legitimacy concerns included the speed at which the vaccines were produced and approved (too fast) and the potentially disingenuous role the government might have played.

Contrary to widespread views that hesitancy was the main barrier among the unvaccinated, we found that perceived structural barriers around access played a more prominent role in our sample. However, two participants mentioned opposing getting vaccinated.

We also identified several themes related to facilitators than enabled participants to get vaccinated, which again focused on vaccine access and social support to motivate them to get vaccinated. More than half of the participants referred to the encouraging, enabling, and supportive roles of specific community-based organizations (CBOs) and individual community leaders (such as church leaders and neighbors involved in non-profits). Participants who had connections to a community group reported more straightforward navigation to get the vaccine because of the relationship. Other enablers included outreach from clinics, language and cultural concordance of information, and vaccine sites.

### Linking Barriers to Intervention Functions and Policy Categories

**Fig. 1:**
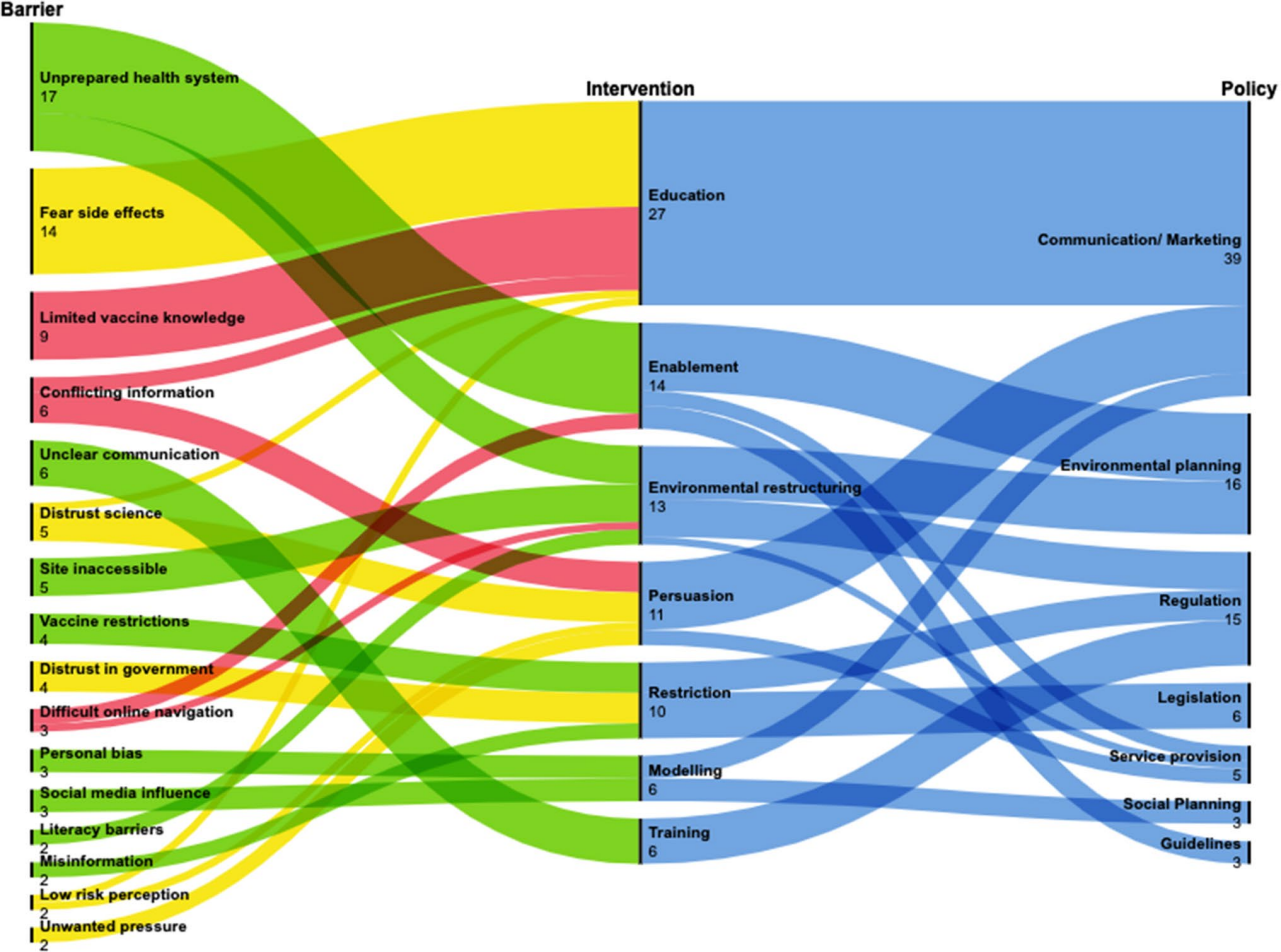
Alluvial chart linking main barriers for COVID-19 vaccine uptake to recommended intervention and policies

Figure 1 maps how the barriers identified through our interviews and described in Table 2 link to intervention functions and policies. The alluvial chart shows the identified barriers color-coded by their COM-B category. Each barrier flows to its corresponding intervention function, and each intervention function to a policy category. The distilled data set was imported to RAWGraphs, an open-access data visualization application to create an alluvial chart. An alluvial chart is a flow chart that helps identify patterns and trends, data categories, and rankings. Variables are assigned to nodes in the parallel columns. Each node represents values ordered in descending order based on their frequency; it shows observations with a stream flowing through the nodes. Alluvial charts are read from left to right, and the size of the vertical nodes (black line) and the stream's width are proportional to the frequency, also shown numerically. The chart shows, from left to right, the identified vaccine uptake barriers, the potential intervention functions and the policies to consider to address the barriers. Barriers in the first column are the same as those discussed in Table 2. The barrier colors represent their COM-B categories, red for capability, green for opportunity, and yellow for motivation. Barriers were only counted once for each participant, even if they came up more times during the interviews.

For example, looking at the first barrier, “unprepared health system,” we can see that all participants (*N* = 17) mentioned it; this is green given that it is an opportunity barrier under COM-B. The stream then flows to the intervention functions, identified through the BCW, that would be adequate for this barrier, in this case, enablement and environmental restructuring; lastly, the stream flows to different policy categories that would support the delivery of these interventions, including environmental planning, service provision, and guidelines.

The main barriers (Column 1) that participants faced the most to access a COVID-19 vaccine were an unprepared health system (*n* = 17), fear of side effects (*n* = 14), limited vaccine knowledge (*n* = 9), and conflicting information (*n* = 6). The COM-B category to which the most barriers belonged was physical opportunity which refers to the opportunity afforded by the environment to get vaccinated; unprepared health system, unclear communication, inaccessible vaccine sites, and literacy barriers were all part of this category. Automatic motivation and psychological capability barriers were also important categories. Fear of side effects was the most significant contributor to automatic motivation, while limited vaccine knowledge and conflicting information made up psychological capability. The intervention functions (column 2) to which the most barriers were linked were education, followed by enablement and environmental restructuring. Lastly, the most relevant policy considerations (column 3) included communication and marketing, environmental planning, and regulation.

## Discussion

The COVID -19 pandemic has disproportionally affected racial and ethnic minorities in the United States, these groups may face additional challenges to adhere to prevention measures and public health programs should be specifically tailored to impacted communities. In partnership with the local public health department’s contact tracing program, we used the COM-B model and BCW framework to understand participants' adherence to COVID-19 prevention measures and identify intervention functions and policies to increase their uptake. At the time of the study, Spanish-speaking Latinx residents were disproportionally infected by COVID-19 and represented most of the contact tracing program’s participants. Using the COM-B model, we used qualitative interviews to identify barriers and enablers for COVID-19 vaccination. Our findings highlight that multiple, often related barriers existed during the initial months of the COVID-19 vaccine roll-out in San Francisco. The behavioral analysis identified that physical opportunity was our participants' most common COM-B category of COVID-19 vaccine uptake barriers. Lack of health system preparedness for assisting a diverse range of non-English speaking patients, inadequate risk communication for Spanish speakers, and limited health literacy in Spanish and English, were significant barriers Spanish Speakers, a high-risk population, faced to get a COVID-19 vaccine. Using the BCW framework we identified that interventions functions to tackle these inter-related barriers, include education, enablement, and environmental restructuring and policies should center around communication and education. Lastly, we found that implementation science frameworks can be used to design and improve public health interventions in real-time.

Our finding that physical opportunity was the most common COM-B category, contradicts other studies that have found automatic motivation to be more common (Liu & Liu, 2021). We did our study in the context of the initial vaccine roll-out, which might explain this difference as there were many incumbrances faced by our sample in terms of access to health care in general. More current reports have found motivational barriers to be the main drivers of not getting vaccinated. Participants in our study perceived that the roll-out of COVID-19 vaccines in San Francisco, as in other places, was confusing due to strict eligibility criteria and a lack of clear communication; in trying to control who got the vaccine, many people were missed or discouraged. Consistent with other studies, we found that systems relying on technology for information and scheduling were at odds with high-risk groups' limited general literacy and tech literacy (Alismail & Chipidza, 2021; Heilweil, 2021; McClain et al., 2021). This is also true in a study in San Francisco among a similar population as ours (Stern et al., 2021). Akin to other studies on ethnic minorities, we found that communication strategies that address the specific communities through education, persuasion, and modeling should be policy priorities (Cassidy et al., 2021; Castillo et al., 2021). Additionally, as others have found, these interventions are better delivered by or in partnership with local CBOs (Eissa et al., 2021; Finney Rutten et al., 2021).

Based on our findings, we suggest the following intervention and policy strategies for improving vaccine uptake, with a focus on strategies that health departments can pursue:

1. Create language and cultural concordant communication campaigns which cover education on the vaccine, science, side effects, the approval process, and education on how and when to get the vaccine in their specific context, which should be persuasive and include behavior modeling.
2. Provide risk communication training for public health professionals, vaccine outreach workers, and community-based organizations working in vaccine outreach.
3. Increase social media reach and investment to create tailored campaigns that promote vaccination through various multi-lingual educational sources (by public health departments and others they work with who are doing vaccine outreach).
4. Work with legislators to regulate the spread of misinformation on social media.
5. Develop a network of vaccine providers that are connected and in close communication with health officials so that any of them can provide information and resources of alternative venues.
6. Create broad vaccine eligibility criteria.
7. Design vaccine sites that are accessible for people with disabilities, literacy barriers, and limited English proficiency.
8. Build solid and equal partnerships with community-based organizations (CBOs) that go beyond the COVID-19 pandemic and leverage these partnerships for public health interventions.
9. Invest in CBOs.

The importance of using behaviorally informed strategies in COVID-19 vaccine campaigns had been highlighted even before vaccines were available (Volpp et al., 2021; Williams et al., 2020). Since then, multiple groups, including ours, have found that implementation science frameworks provide the template to achieve this. Similar to their research, we found that these frameworks can be used to tailor the response to specific at-risk populations, ensuring a more equitable pandemic response (Lee et al., 2021; Vallis et al., 2021). This is the first publication, to our knowledge, to apply the COM-B model to understand barriers within a public health COVID-19 contact tracing program. As a result, we believe it provides important insights for health departments beyond the scope of COVID-19 prevention. Our findings highlight the existing vulnerabilities and social inequities that exist within ethnic and racial minorities in the United States. Most of the personal level barriers we identified are directly related to preexisting forms of discrimination in our studied population, including poverty, housing insecurity, and low levels of literacy and education (Nguyen et al., 2021). These characteristics increase their risk of getting COVID-19 and the challenges of getting a vaccine. There is a need to research and develop interventions that account for the intersectionality of risk factors in this group (Shapiro et al., 2021).

This study is limited by the small sub-set of interviews conducted throughout the pandemic. However, due to the targeted sampling approach, we could reach thematic saturation with the included participants. With a focus on San Francisco residents, it is unclear how generalizable the findings are. San Francisco has more resources than other cities, and Spanish speakers might face different barriers in other places. Due to the changing nature of the pandemic, our findings might not be reflective of the pandemic over time and might be less salient now than they were a few months ago. Additionally, we only included participants who agreed to be part of the SF-DPH contact tracing program, which could lead to selection bias by having only participants willing to engage in other COVID-19 public health activities. However, the overall program participation rate was high, and as a result, we believe this to be a minor limitation. The purpose of our project was to give policymakers recommendations for program improvement; we did not implement or measure the impact of the intervention and policy proposals. Despite these limitations, our paper suggests how public health departments and academic institutions can work together to bridge the gaps between research and implementation. Most existing implementation science on vaccine uptake focuses on identifying barriers or intervention design outside of an existing program; a significant strength of our project is that it was conceived as an embedded study within an existing public health program and used to identify barriers and solutions in real-time, facilitating implementation. Our sampling approach allowed us to identify the rich diversity of experiences within a sample of the most highly impacted people in San Francisco, which were disproportionately Spanish speakers; we used this unique sampling approach to collect a real-time sample among those in the contact tracing program.

Our findings suggest that the COM-B model and BCW framework can be part of public health programs and provide real-time evidence on how to incorporate human behavior into interventions in a rapidly evolving situation, as with a pandemic. Our alluvial chart shows what intervention functions and policy categories stakeholders should focus on to increase vaccine uptake within this population in San Francisco and was broadly shared through presentations and reports. Additionally, we show how an academic-public health partnerships can be leveraged in pandemic response and used to improve and design interventions in real-time. Our study results were shared regularly with the SFDPH, and the final findings were disseminated to external stakeholders in other California counties and the California Department of Public Health.

There is no one-size-fits-all approach to public health interventions. Public health departments must tailor the response to each community or sub-population by first understanding the specific barriers they might face. Our research suggests that implementation science can provide frameworks for public health interventions to incorporate behavior into their design in a ‘real-time’ flexible way and help develop adjustments in policy and practice, to ensure the public health response is equitable. This project was a partnership between UCSF researchers and the SFDPH to ensure the COVID-19 response reached the Spanish-speaking population. Future research should focus on how people overcame their perceived barriers and how behavioral and implementation frameworks can be used to plan the roll-out of non-pharmaceutical and pharmaceutical interventions in public health emergencies, such as outbreak and pandemic responses. Our research suggests that incorporating implementation science into public health programs early on can be beneficial. The next steps should include scaling up these strategies and implementing them in broader and more widespread public health programs.
